# Randomized controlled trial investigating web-based, therapist delivered eye movement desensitization and reprocessing for adults with suicidal ideation

**DOI:** 10.3389/fpsyt.2024.1361086

**Published:** 2024-02-16

**Authors:** Lisa Burback, Sidney Yap, Scot E. Purdon, Adam Abba-Aji, Katie O’Shea, Suzette Brémault-Phillips, Andrew J. Greenshaw, Olga Winkler

**Affiliations:** ^1^ Department of Psychiatry, Faculty of Medicine and Dentistry, University of Alberta, Edmonton, AB, Canada; ^2^ Alberta Hospital Edmonton, Edmonton, AB, Canada; ^3^ Heroes in Mind, Advocacy and Research Consortium, Faculty of Rehabilitation Medicine, University of Alberta, Edmonton, Edmonton, AB, Canada; ^4^ Eye Movement Desensitization and Reprocessing International Association, Austin, TX, United States; ^5^ Department of Occupational Therapy, Faculty of Rehabilitation Medicine, University of Alberta, Edmonton, AB, Canada

**Keywords:** eye movement desensitization and reprocessing, suicidal ideation, telepsychiatry, transdiagnostic treatment, psychotherapy, web-based EMDR, clinical trial, trauma focused psychotherapy

## Abstract

**Introduction:**

Promising preliminary evidence suggests that EMDR may reduce suicidal ideation (SI) when used to treat Major Depressive Disorder, Posttraumatic Stress Disorder, and trauma symptoms in the context of acute mental health crises. EMDR has never been tested specifically for treating SI, and there is a lack of data regarding the safety and effectiveness of web-based, therapist-delivered EMDR in populations with known SI. The primary objective of this study was to investigate the impact of web-based, therapist-delivered EMDR, targeting experiences associated with suicidal thinking. Secondary objectives included examining the effect of EMDR treatment on symptoms of depression, anxiety, posttraumatic stress, emotional dysregulation, and dissociation, as well as safety and attrition.

**Methods:**

This randomized control trial (ClinicalTrials.gov ID number: NCT04181047) assigned adult outpatients reporting SI to either a web-based EMDR intervention or a treatment as usual (TAU) group. TAU included primary and mental health services available within the Canadian public health system. Participants in the EMDR group received up to 12 web-based EMDR desensitization sessions, delivered twice weekly during the COVID-19 pandemic (2021-2023). The Health Research Ethics Board at the University of Alberta approved the protocol prior to initiation of data collection for this study (protocol ID number: Pro00090989).

**Results:**

Forty-two adult outpatients received either EMDR (n=20) or TAU (n=22). Participants reported a high prevalence of early onset and chronic SI, and there was a high rate of psychiatric comorbidity. In the EMDR group, median SI, depression, anxiety, and posttraumatic symptom scale scores decreased from baseline to the four month follow-up. In the TAU group, only the median SI and posttraumatic symptom scale scores decreased from baseline to four month follow up. Although sample size precludes direct comparison, there were numerically fewer adverse events and fewer dropouts in the EMDR group relative to the TAU group.

**Conclusion:**

Study results provide promising preliminary evidence that web-based EMDR may be a viable delivery approach to address SI. In this complex population, a short treatment course was associated with reductions of SI and other symptoms across multiple diagnostic categories. Further investigation is warranted to verify and extend these results.

**Clinical Trial Registration:**

https://clinicaltrials.gov/study/NCT04181047?id=NCT04181047&rank=1, identifier NCT04181047

## 1 Introduction

Suicidal ideation (SI) is a marker of severe stress and psychological burden. A history of psychological trauma and adverse childhood experiences (ACEs) is associated with experiencing SI (1). Such stressors are also strongly linked to the development of a range of psychiatric symptoms and disorders, including mood, anxiety, trauma, and personality disorders (2–5). Experiencing four or more types of ACEs is linked with a tenfold increased risk of SI (6). Individuals experiencing SI are likely to present with a wide range of mental health symptoms that may require psychiatric care.

There is a need to expand treatment options for SI, given the recognized limitations of current evidence-based, gold-standard non-pharmacological treatments, which include Cognitive Behavior Therapy (CBT), Dialectical Behavior Therapy (DBT), and Crisis Response Planning. These established interventions are often modestly effective for reducing SI and suicidal behaviors, can be highly resource-intensive, and suffer high client/patient attrition rates (7, 8). Rather than resolving underlying pathogenic memories, these interventions tend to focus on stabilization, cognitive strategies, and coping skills for times of emotional pain or crisis. While that approach fits with the generally accepted and conservative risk management approach for working with patients with complex mental health needs (9, 10), such methods may not aid in the resolution of long-standing, recurrent suicidal states.

Trauma focused psychotherapies (TFPs) hold promise for addressing suicidality (11–15). Interest in using TFPs to treat SI likely stems not only from the observation that trauma is a factor in the development of SI across a wide range of psychiatric disorders, but also because trauma treatment, even when not specifically targeting suicidality, may lead to a reduction in SI (16). PTSD treatment studies often report a reduction of SI along with decreased trauma and depression symptoms (16–18). A recent eye movement desensitization and reprocessing (EMDR) study that targeted trauma-related memories in those experiencing a mental health crisis with SI also demonstrated positive outcomes, with few adverse events (14). EMDR has also been studied prospectively for Major Depressive Disorder (MDD) (19–21), which is the most common mental illness suffered by those who complete suicide (22). Fereidouni et al. reported that nine EMDR sessions administered over 3 weeks significantly reduced SI in depressed inpatients compared to control (12). Such treatment, however, can evoke intense emotions, raising the possibility of worsening or treatment-emergent SI, driving clinician hesitancy to provide such treatments to patients already endorsing such thoughts (23–25). There is also a dearth of studies investigating the safety and efficacy of using remotely delivered EMDR in the context of SI, leaving clinicians with little empirically based guidance.

The Adaptive Information Processing (AIP) model of EMDR posits that traumatic or stressful experiences can overwhelm or otherwise interrupt the brain’s normal capacity to integrate information. When reactivated by trauma reminders, maladaptively stored information can present as psychiatric symptoms (26, 27). Altered states of consciousness that accompany extreme stress, dissociation, drug intoxication or physical illness can set the conditions for altered information processing during and following a stressful event (26–28). Implicit or explicit reminders of such content can reactivate the corresponding distressing affective, sensorimotor, and cognitive information stored in these memories. Survival responses, such as fight, flight, fawn, and freeze, are also believed to be embedded within such memories and can become elaborated over time as traumas accumulate. In such situations, modest stressors may act as reminders of these traumas and evoke a response that is much more intense than expected. The person may consequently re-experience states similar to those experienced at the time of the trauma, along with the same emotions, sensations, beliefs, defensive postures, urges, reflexes, and stress responses. As much of this information is stored in implicit, non-declarative memory, the person may not be aware that a memory is being activated; it is therefore experienced as happening *now*. Depression and trauma-related shame, for example, have been conceptualized as implicit memory networks constructed from previous experiences of shame, humiliation, and loss (29, 30). Thus, a mild perceived rejection might activate a network of unconscious memories, subjectively experienced as intense shame, physiological distress, and a strong urge to hide, attributed to the present situation. Access to healthy, adaptive information, held in other networks, can be simultaneously restricted (26). Resulting feelings and behaviors may be inconsistent with the person’s present developmental level or circumstances.

The AIP model postulates that EMDR addresses such *maladaptively stored* or *pathogenic memories.* It is hypothesized that EMDR reduces the negative valence of these memories and allows adaptive information to be integrated into the associated brain networks, thereby promoting a more balanced and constructive emotional response. Consequently, reminders no longer evoke distress or the previously rigidly held maladaptive beliefs (26, 30, 31). The AIP model aligns with neurobiological research indicating how alterations in memory formation, conditioning, learning, and memory reconsolidation processes contribute to the development of a wide range of psychiatric disorders (26, 32–35). Additionally, this perspective is consistent with studies demonstrating that brain areas impacted by early life stress during development are also those involved in memory, emotion and emotional regulation, self-referential processing (e.g., Default Mode Network), sensation and information integration (e.g., limbic system, prefrontal cortex, thalamus, insula, and brainstem) (28, 36–39), and that EMDR or bilateral eye movements influence many of these same brain areas (40–43).

The potential of using the AIP model to understand the roots of suicidal thinking emerges when comparing existing suicide theories against the AIP model (**Table 1**). Suicide theories generally attribute SI to repetitive distressing states arising from aversive experiences and their consequences, such as the development of suicide-related beliefs. The Interpersonal Theory of Suicide and the Integrated Motivational-Volitional Model, for example, emphasize interpersonal experiences such as those arising from rejection, isolation, defeat, and the perception of being trapped or a burden on others (47, 48). Most theories suggest that suicide attempts occur once a person develops the capacity to act on suicidal thoughts, perhaps due to habituation to the aversion of acting on such impulses, perception of lack of alternative options, or a desire to escape from intolerable pain, loneliness, defeat, entrapment, burdensomeness, or hopelessness (49).

**Table 1:** Brief description of major suicide theories (44–46).

Suicide Theory	Main Ideas
Interpersonal Theory of Suicide (IPTS)	• Suicide attempts result from suicidal desire plus capacity to act • Suicidal desire arises from thwarted belongingness and perceived burdensomeness • Repeated painful events allow habituation to fear of suicide
Escape Theory	• Stressful events activate painful affective states • Leads to escape urges, reduced inhibition, increased negative and suicidal thinking • Over time, barriers to suicide diminish; suicide becomes the solution to inescapable, intensely negative states
Fluid Vulnerability Theory	• Distressing events lead to development of suicidal “states” and belief systems • External or internal triggers activate these states • Suicidal states have characteristic cognitive (e.g., unlovability, helplessness, burdensomeness), affective, behavioral, and physiological (e.g., arousal, sensations) aspects
Integrated Motivational-Volitional Model	• Like IPTS, but emphasizes defeat and entrapment rather than belongingness and burdensomeness
Three-step Theory	• Step 1: Pain and hopelessness leads to SI • Step 2: SI escalates when pain overwhelms connectedness • Step 3: Strong suicidal desire progresses to suicide attempts if capability for suicide is present

The Fluid Vulnerability Theory (FVT) most closely mirrors the AIP model. Originally based on Beck’s notion of “integrated cognitive-affective-behavioral networks” producing a “synchronous response to external demands”, the FVT suggests that past painful events lead to the development of suicidal states that are triggered by reminders (50, 51). Such states are conceptualized to include not only emotional and cognitive components, but also sensorimotor and physiological information (e.g., urges, sensations, arousal), echoing the AIP model of unprocessed, negatively valenced neural networks. As such, we hypothesized that targeting experiences, beliefs, and affective states associated with SI with EMDR would reduce the intensity of those states. We further theorized that beliefs would shift in a “bottom up” manner once the memories were desensitized, enabling the integration of more adaptive information from other experiences. For a deeper exploration of this hypothesis, refer to Winkler et al. (1). This perspective is also consistent with recent calls to view suicidality as a manifestation of adaptive processes gone awry, best studied through a neuroscience approach like the Research Domain Criteria (RDoC) framework, rather than imprecise “verbal theories” of suicide (49).

The primary objective of this study was to investigate whether web-based, synchronous clinician-delivered EMDR, primarily targeting experiences, beliefs or affective states associated with suicidal thinking, would aid in reducing SI compared to treatment as usual (TAU). Secondary objectives included examining the impact of web-based EMDR treatment on symptoms of depression, anxiety, posttraumatic stress, emotional dysregulation, and dissociation, as well as treatment safety and attrition.

## 2 Materials and methods

This pilot study is a real-world non-blinded randomized controlled trial that aimed to evaluate the effects of web-based clinician-delivered synchronous EMDR for adult outpatients with SI. Those enrolled in the study were randomly assigned to either the EMDR treatment or TAU group using the randomization module of REDCap (Research Electronic Data Capture) (52).

Approval by the Health Research Ethics Board at the University of Alberta was obtained prior to commencing research activities (protocol ID number: Pro00090989). This study was registered on ClinicalTrials.gov (ID number: NCT04181047). For detailed information regarding the study protocol, rationale, and clinical safety procedures used, refer to Winkler et al. (1).

### 2.1 Participant recruitment and eligibility

Participants were recruited from publicly funded adult health services serving individuals aged 18-65 years in and around Edmonton, Alberta, Canada, as well as through a self-referral website. Mental health services providing referrals included crisis and mental health triage referral services, Edmonton area hospitals, community-based addiction and mental health services, private practice, primary care, and the city day hospital, which serves as an alternative to hospitalization. After initial screening, participants underwent a psychiatric assessment and diagnostic interview with one of two study psychiatrists to assess eligibility prior to enrollment in the study. Inclusion criteria included being between 18-65 years of age, reporting SI in the past week, having an established and ongoing relationship with another healthcare provider outside the study, having access to appropriate technology and space for online therapy (e.g., a working computer and a private space for sessions), and committing to twice weekly EMDR sessions for the study duration. Exclusion criteria included severe dissociative symptoms or a dissociative disorder, determined either through the diagnostic assessment, or a Dissociative Experiences Scale II (DES-II) score above 34. Examples of severe dissociative symptoms meeting exclusion criteria included dissociative voices, amnestic episodes, dissociative fugue states, passivity experiences, first rank symptoms under stress, subjective experiences of alter personality self-states, and severe alexithymia associated with inability to feel body sensations or attune to emotions. Those with a history of mania or psychosis in the absence of substance use, those receiving concurrent electroconvulsive therapy (ECT) or another TFP during the study period, and pregnant individuals were also excluded.

We intentionally chose to recruit participants based on SI and not a specific diagnosis (see Winkler et al. (1)) to test the hypothesis that EMDR could act as a transdiagnostic treatment for SI by addressing implicit and explicit memories prompting or promoting suicidal ideation (26, 27). Suicidal ideation crosses diagnostic boundaries, and psychological theories explaining the basis of suicide are transdiagnostic (44–46). Further, clinicians face the dilemma that although many suicidal patients carry multiple diagnoses, such complex patients are often excluded from clinical trials to avoid heterogeneity that could obscure statistically significant study results. We chose to risk a negative result in favor of enrolling a real-world sample that would increase clinical applicability of the results.

### 2.2 Treatment groups

#### 2.2.1 EMDR treatment group

EMDR participants received 12 sessions of therapist-delivered, web-based EMDR, delivered twice weekly via secure Zoom video conferencing; they also had access to treatment as usual. Therapists in this study were EMDR International Association (EMDRIA) certified psychiatrists trained in multiple therapy modalities. Prior to commencing EMDR sessions, participants received psychoeducation about EMDR, developed a safety plan with the therapist, and participated in up to five preparation exercises (1). Therapists developed individualized case conceptualization and treatment plans, which included identifying treatment targets associated with SI; these were used in the subsequent 12 EMDR sessions. The standard EMDR protocol was generally used, with modifications allowed if study therapists deemed these to be clinically necessary. For further details regarding EMDR procedures utilized, see Winkler et al. (1).

#### 2.2.2 TAU group

TAU group participants received primary and mental health services available without charge within the Canadian public health system, provided these treatments did not meet exclusion criteria (e.g., no ECT or another TFP). This included routine visits for mental health evaluation and/or medication adjustment with a psychiatrist or family doctor, and/or visits with a mental health therapist. Mental health clinics generally assign a mental health therapist as a case manager, who provides counseling, psychotherapy, psychiatrist consultation, or referral to other services as clinically needed. Typical available interventions include crisis services, supportive, solution-focused, CBT, DBT or psychodynamic based interventions.

### 2.3 Primary and secondary outcome measures

Study psychiatrists conducted a baseline assessment with all participants, including a clinical psychiatric interview, which established Diagnostic and Statistical Manual of Mental Disorders-5 (DSM-5) based diagnoses (53), and a clinician-rated Columbia Suicide Severity Rating Scale (CSSRS) (54).

Participants also completed several primary and secondary self-rated outcome measures at baseline. Primary outcome measures for SI included the Beck Scale for Suicide Ideation (BSS) and the 5-item CSSRS-last week. The BSS is a 21-item questionnaire reporting on SI and suicidal behavior over the past week. Scores range from 0 to 42, with higher scores indicating worse outcomes (digital adaptation 2021 NCS Pearson Inc. All rights reserved. Adapted and used under license #LSR-262494) (55). The CSSRS self-rated is a self-report version of the CSSRS clinician-rated assessment. It consists of 5 questions on SI (score range 0 to 5), as well as a question asking about the presence or absence of any preparatory or actual suicidal behavior during two time periods (lifetime and last three months) (54).

Secondary outcome measures included the Beck Depression Inventory-II (BDI-II) and Patient Health Questionnaire-9 (PHQ-9) for depression, Generalized Anxiety Disorder-7 (GAD-7) for anxiety, Impact of Events Revised (IES-R) for trauma related symptoms, DES-II for dissociation, Difficulties in Emotional Regulation Scale (DERS) for emotion dysregulation, and the self-reported Adverse Childhood Experiences Scale (ACES). The BDI-II is a 21-item questionnaire (score range 0 to 63), with higher scores indicating a higher likelihood of MDD (digital adaptation 2021 NCS Pearson Inc. All rights reserved. Adapted and used under license #LSR-262494) (56). The PHQ-9 is a self-report questionnaire for assessing depressive symptoms during the previous 2 weeks, with higher scores (score range 0 to 27) indicating more severe depressive symptoms (57). The GAD-7 is used to measure severity of anxiety symptoms over the past 2 weeks, with higher scores indicating worse symptoms (score range 0 to 21) (58). The IES-R rates the intensity of distress and PTSD-like symptoms related to a past event over the past 7 days, with higher scores (range 0 to 88) indicating more severe symptoms (59). The DES-II includes 28 items about state and trait dissociative symptoms, each scored from 0 to 100 based on the frequency of experiencing the symptom. An average score is calculated, with a higher score indicating more severe dissociative pathology. A previous study reported that the mean DES-II scores for PTSD, Dissociative Disorder Not Otherwise Specified, and Dissociative Identity Disorder were 31, 36, and 48, respectively (60). The DERS scale reports on symptoms related to emotion regulation, where participants rate how often the statements presented in each of the 36 items apply to them (score range 36 to 180) (61). The ACES is a standard 10-item questionnaire that assesses the presence or absence of adversities experienced in the first 18 years of life. Higher scores are indicative of more childhood adversity (62).

All participants completed primary and secondary outcome self-report measures again at two and four months after baseline measures were completed. Exceptions included the ACES, which was only completed at baseline, and the DES-II and DERS, which were only repeated at the four-month time point. All study data was collected and stored using REDCap (52).

Patient safety data, including completed suicides, suicide attempts, emergency room visits, hospitalizations, and dropouts, were also tracked. Emergency room visits and hospitalizations were obtained from Alberta’s provincial public electronic health record system.

### 2.4 Data analysis

Collected data was first de-identified and then analyzed using IBM SPSS Statistics software (Version 28.0) (63). Descriptive statistics were calculated for the EMDR and TAU groups. An intention to treat analysis was completed, wherein data from all EMDR and TAU group participants were included for data analysis. In the event of measure non-response, median scores were used as a placeholder value. Non-parametric analyses were conducted due to the relatively small sample size of each participant group. A conservative approach was used, correcting for multiple comparisons, to reduce type 1 error. A Friedman’s test was used to assess for significant differences (p≤ 0.05) over time. A follow-up Wilcoxon signed rank test was used with a Benjamini-Hochberg correction to delineate specific *post-hoc* comparisons between time points. The demographic and baseline clinical severity data was compared between groups to check for differences which may have led to relevant variance in outcome measure scores.

## 3 Results

### 3.1 Demographics

**Figure 1:**
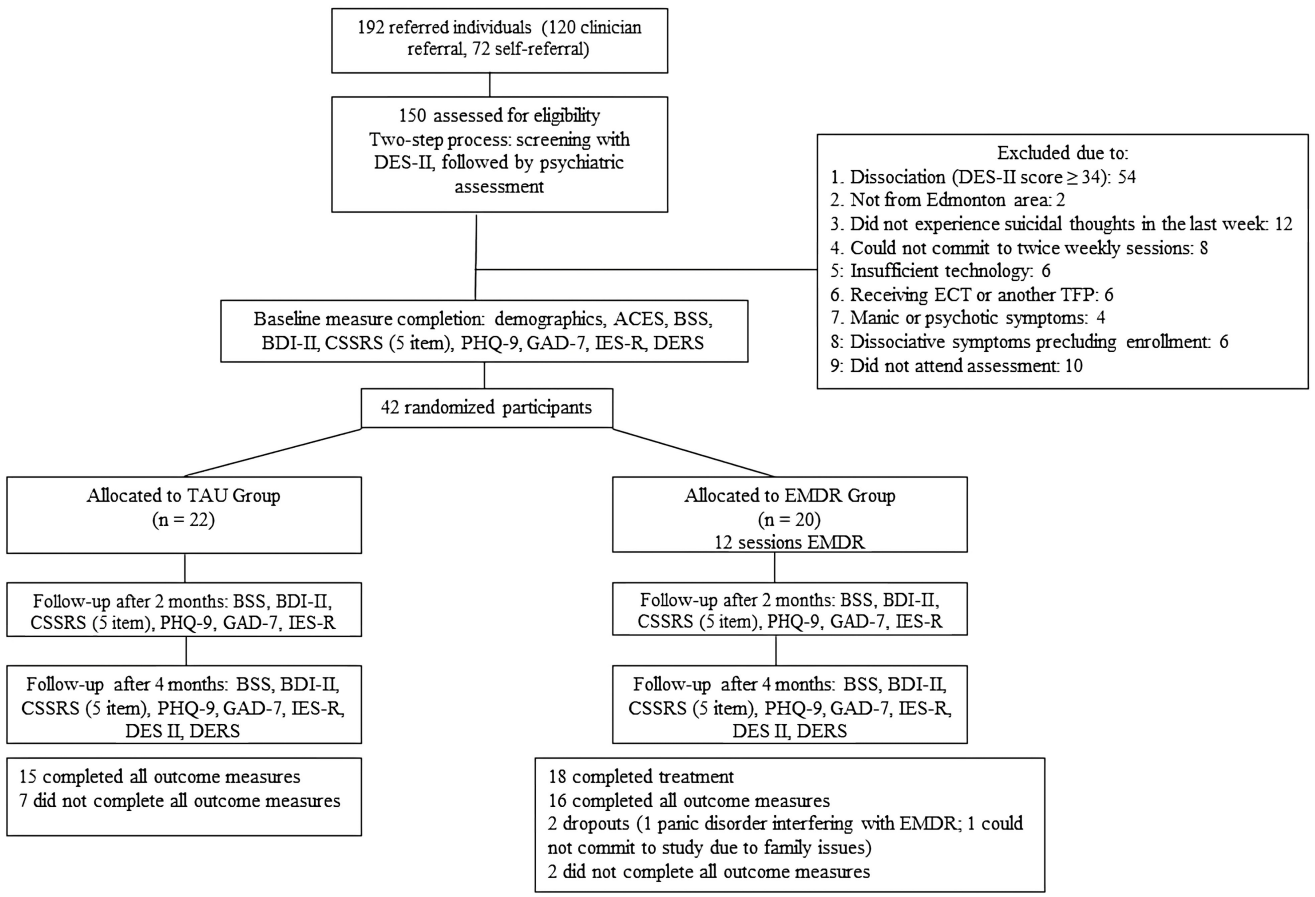
Flow of the study participants.

Overall, 42 adult outpatients were enrolled in the study, with 20 randomized to the EMDR group and 22 to the TAU group (see **Figure 1**). EMDR group participants self-identified as female (n=14), or male (n=6), all aligning with their sex at birth. TAU group participants self-identified as women (n=19), men (n=3), female (n=17), male (n=3), non-binary (n=1), or transgender (n=1). The average participant age was 36 years in both groups. Referral services included self-referral (n=12), day hospital (n=12), outpatient addiction, mental health, or primary care services (n=11), crisis and triage referral services (n=5), and Edmonton area hospitals (n=2). See **Table 2** for a full summary of participant baseline socio-demographic characteristics.

**Table 2:** Participant baseline socio-demographic and psychiatric characteristics.

	EMDR Group (n=20)	TAU Group (n=22)	All Participants (n=42)
Socio-demographic Characteristics			
Sex	Women: 14 (70%) Men: 6 (30%)	Women: 19 (86%) Men: 3 (14%)	Women: 33 (79%) Men: 9 (21%)
Gender	Female: 14 (70%) Male: 6 (30%)	Female: 17 (77%) Male: 3 (14%) Non-Binary: 1 (4.5%) Transgender: 1 (4.5%)	Female: 31 (74%) Male: 9 (22%) Non-Binary: 1 (2%) Transgender: 1 (2%)
Mean Age ± SD	36 ± 12	36 ± 12	36 ± 12
Sexual Orientation	Heterosexual: 11 (55%) Bisexual: 7 (35%) Asexual: 1 (5%) Prefer Not to Say: 1 (5%)	Heterosexual: 14 (64%) Bisexual: 4 (18%) Homosexual: 2 (9%) Prefer Not to Say: 2 (9%)	Heterosexual: 25 (60%) Bisexual: 11 (26%) Homosexual: 2 (5%) Asexual: 1 (2%) Prefer Not to Say: 3 (7%)
Ancestry or Race	Caucasian: 13 (65%) Multiracial: 4 (20%) Asian: 1 (5%) Black: 1 (5%) Indigenous: 1 (5%)	Caucasian: 16 (72%) Multiracial: 4 (18%) Asian: 1 (5%) Prefer Not to Say: 1 (5%)	Caucasian: 29 (70%) Multiracial: 8 (19%) Asian: 2 (5%) Black: 1 (2%) Indigenous: 1 (2%) Prefer Not to Say: 1 (2%)
Relationship Status	Married or Partnered: 13 (65%) Separated or Divorced: 4 (20%) Single: 3 (15%)	Married or Partnered: 11 (50%) Separated or Divorced: 1 (5%) Single: 9 (40%) Widowed 1 (5%)	Married or Partnered: 24 (57%) Separated or Divorced: 5 (12%) Single: 12 (29%) Widowed 1 (2%)
Highest Completed Level of Schooling	4-year degree: 3 (15%) Some college: 10 (50%) High School: 3 (15%) Up to Grade 12: 4 (20%)	4-year degree: 8 (36%) Some college: 10 (46%) High School: 4 (18%)	4-year degree: 11 (26%) Some college: 20 (48%) High School: 7 (17%) Up to Grade 12: 4 (9.5%)
Employment Status	Full-time: 7 (35%) Part-time: 6 (30%) Unemployed: 7 (35%)	Full-time: 10 (45%) Part-time: 2 (10%) Unemployed: 10 (45%)	Full-time: 17 (40%) Part-time: 8 (20%) Unemployed: 17 (40%)
Receiving Disability Benefits	Yes: 6 (30%) No: 14 (70%)	Yes: 11 (50%) No: 11 (50%)	Yes: 17 (40%) No: 25 (60%)
Baseline Psychiatric Characteristics			
Trauma-related Disorder	9 (45%)	9 (41%)	18 (43%)
Borderline Personality Disorder	6 (30%)	9 (41%)	15 (36%)
Strong BPD Traits	5 (25%)	6 (27%)	11 (26%)
Personality Disorders or Traits OTHER than BPD	6 (30%)	3 (14%)	9 (21%)
Mood Disorder	18 (90%)	19 (86%)	37 (88%)
Anxiety Disorder	14 (70%)	7 (32%)	21 (50%)
Substance Use Disorder	2 (10%)	2 (9%)	4 (10%)
3 or More Concurrent Diagnoses	15 (75%)	11 (50%)	26 (62%)
Age of Onset for Suicidal Ideation	Mean: 14 Median: 13 Range: 7 to 50	Mean: 14 Median 14 Range: 6 to 27	Mean: 14 Median: 13.75 Range 6 to 50
History of Non-suicidal Self-injury	Yes: 14 (70%) No: 6 (30%)	Yes: 11 (50%) No: 11 (50%)	Yes: 25 (60%) No: 17 (40%)
Suicide Related Hospitalizations (Lifetime)	Mean: 0.87 Median: 0 Range: 0 to 6	Mean: 0.98 Median: 0 Range: 0 to 6	Mean: 0.95 Median: 0 Range: 0 to 6
Suicide Attempts (Lifetime)	Mean: 2 Median: 1 Range: 0 to 20	Mean: 2 Median: 1 Range: 0 to 10	Mean: 2 Median: 1 Range: 0 to 20

### 3.2 Baseline psychiatric characteristics

The study population was complex, with a high rate of psychiatric comorbidity and high incidence of early onset and longstanding SI. Eighty-eight percent of respondents met criteria for a mood disorder, 43% met criteria for a trauma-related disorder, usually PTSD, and 62% had BPD or strong BPD traits. Three quarters of the EMDR group had at least three DSM 5 diagnoses. The median age of first experiencing SI was 13 and 14 years of age in the EMDR and TAU group, respectively (range 6 to 50 years). Median prior suicide attempts were 1 in both groups (EMDR: range 0 to 20; TAU: range 0 to 10). Baseline median BSS scores were 16.5±8.06 (range 0 to 25) and 20±7.60 (range 3 to 31) in the EMDR and TAU group, respectively. See **Table 2** for a full summary of participant baseline psychiatric characteristics.

### 3.3 Primary outcomes

Of the 20 participants in the EMDR group, 18 received all 12 web-delivered sessions. Of these 18 participants, 4 completed their desensitization sessions prior to completing their midpoint measures. The median number of desensitization sessions completed prior to completing midpoint measures was 8 (range 4 to 12). There were two dropouts: 1 participant with panic disorder could not tolerate feeling bodily sensations, which interfered with EMDR; 1 participant dropped out due to family issues.

Friedman’s test indicated significant differences in BSS and CSSRS scores across all three time points (BSS: p=0.001; CSSRS: p=0.016) for EMDR participants. Additional analysis using the Wilcoxon comparison, with Benjimani-Hochberg correction, indicated significant differences in median BSS scores between baseline and endpoint (Z=-2.617, p=0.009) and midpoint and endpoint (Z=-2.164, p=0.03), as well as significant differences in median CSSRS scores between baseline and endpoint (Z=-2.908, p=0.004).

TAU group participants reported significant differences in BSS scores across all three time points using the Friedman’s test (BSS: p=0.05). Further analysis using the Wilcoxon comparison, with Benjimani-Hochberg correction, indicated significant differences in median BSS scores between baseline and endpoint (Z=-2.596, p=0.009). See **Table 3** for a full summary of outcome measure scores.

**Table 3:** Primary and secondary outcome measure median scores in EMDR (n=20) and TAU (n=22) groups.

Outcome Measure	EMDR Group Self-report Measures			TAU Group Self-report Measures		
	Baseline Score±SD	Midpoint Score±SD	Endpoint Score±SD	Baseline Score±SD	Midpoint Score±SD	Endpoint Score±SD
Suicidality scales						
BSS	16.5±8.1	14±7.8	12±7.2* (p=0.009)	20±7.6	16±9.5	13±9.6* (p=0.009)
CSSRS-self-rated	3±1.4	3±1.3	1.5±1.1* (p=0.004)	3±1.4	2±1.5	3±1.5
Symptoms related to past stressful events						
IES-R	33±12.4	23±14* (p=0.003)	12±19* (p=0.002)	49.5±17.4	27±22.8* (0.005)	37±22* (0.007)
Depression						
BDI-II	35±10.6	26±13.7* (p=0.001)	19±15.7* (p=0.005)	41.5±8.7	31±15.2	40±14.6
PHQ-9	21±6.5	16±6.5* (p=0.039)	12.5±7.1* (p=0.001)	22±4.7	17.5±7.5* (p=0.033)	22.5±7.5
Anxiety						
GAD-7	14.5±4.9	11±5.3* (p=0.001)	8.5±5.8* (p=0.004)	16±5.2	12±6.4	14±5.9
Dissociation						
DES-II	14.6±7	N/A	11.6±7.8	17.9±7.3	N/A	13.4±12
Emotion regulation						
DERS	108±17.9	101±18.7	95±22.1	123±16	115.5±28.4	117±20.8

DES II, Dissociative Experiences Scale; BSS, Beck Scale for Suicide Ideation; BDI-II, Beck Depression Inventory II; CSSRS, Columbia Suicide Severity Rating Scale; PHQ-9, Patient Health Questionnaire 9; GAD-7, Generalized Anxiety Disorder 7; IES-R, Impact of Events Revised; DERS, Difficulties in Emotional Regulation Scale. *=significant difference from baseline (p≤ 0.05 corrected for multiple comparisons).

### 3.4 Secondary outcomes

EMDR group participants reported significant differences in BDI-II (p=0.001), PHQ-9 (p=0.023), GAD-7 (p=0.014), and IES-R (p=0.001) scores across all three time points. No significant differences were found in DERS or DES II scores. Further analysis showed significant differences in baseline and midpoint BDI-II, PHQ-9, GAD-7, and IES-R scores, baseline and endpoint BDI-II, PHQ-9, GAD-7, and IES-R scores, and midpoint and endpoint BDI-II and PHQ-9 scores.

TAU group participants reported significant differences in PHQ-9 (p=0.006) and IES-R (p=0.011) scores across all three time points. No significant differences were found in BDI-II, GAD-7, DERS, and DES-II scores. Further analysis showed significant differences in baseline and midpoint PHQ-9 and IES-R scores, baseline and endpoint IES-R scores, and midpoint and endpoint PHQ-9 scores. See **Table 3** for primary and secondary outcome measure scores and **Supplementary Table 1** for a summary of Wilcoxon comparisons. Serious adverse events are reported in **Table 4**.

**Table 4:** Serious adverse events reported in EMDR (n=20) and TAU (n=22) groups.

Adverse Event	EMDR Group	TAU Group
Completed Suicide	0	0
Suicide Attempts	1	5
Psychiatric Emergency Room Visits	2	5
Psychiatric Hospitalizations	0	2

In summary, both EMDR and TAU group participants reported significant differences in suicide scale scores between baseline and endpoint. EMDR group participants also reported significant differences in depression, anxiety, and posttraumatic symptom scale scores between baseline and endpoint, while TAU group participants only reported significant differences in posttraumatic symptom scale scores between baseline and endpoint. EMDR group participants reported fewer serious adverse events compared to TAU group participants.

## 4 Discussion

This study is unique and represents a significant contribution to the literature at several points. First, the study was originally conceived based on the AIP model and the hypothesis that EMDR could directly target the “roots” of suicidality. SI was therefore an inclusion criterion and therapists specifically targeted experiences associated with SI rather than a specific diagnosis. Second, it included a complex, real-world psychiatric population. At the time this study was initiated, many would have considered it too risky to focus on such distressing material. Studies of this nature are infrequently conducted given the perceived risks, including adverse events, dropouts, and the potential for negative results due to participant heterogeneity. Third, this study is among the first to report on remote delivery of EMDR in a real-world context, which was infrequent before the pandemic.

Study results were favorable, although methodological factors may have impacted outcomes. Both the EMDR and TAU care groups exhibited a decrease in self-reported SI and PTSD-like symptoms from baseline to four month follow up. The EMDR group experienced an additional significant decrease in depression and anxiety symptoms over the same time frame, a change that was not observed in the TAU group. Although the small sample size limits data interpretation, there appears to be a downward trend in DERS and DES-II scores in the EMDR group as well. Far from worsening symptoms, EMDR treatment focused on SI resulted in ongoing improvement across multiple symptom categories. Larger studies are needed to assess the reliability of this observation.

**Figure 2:**
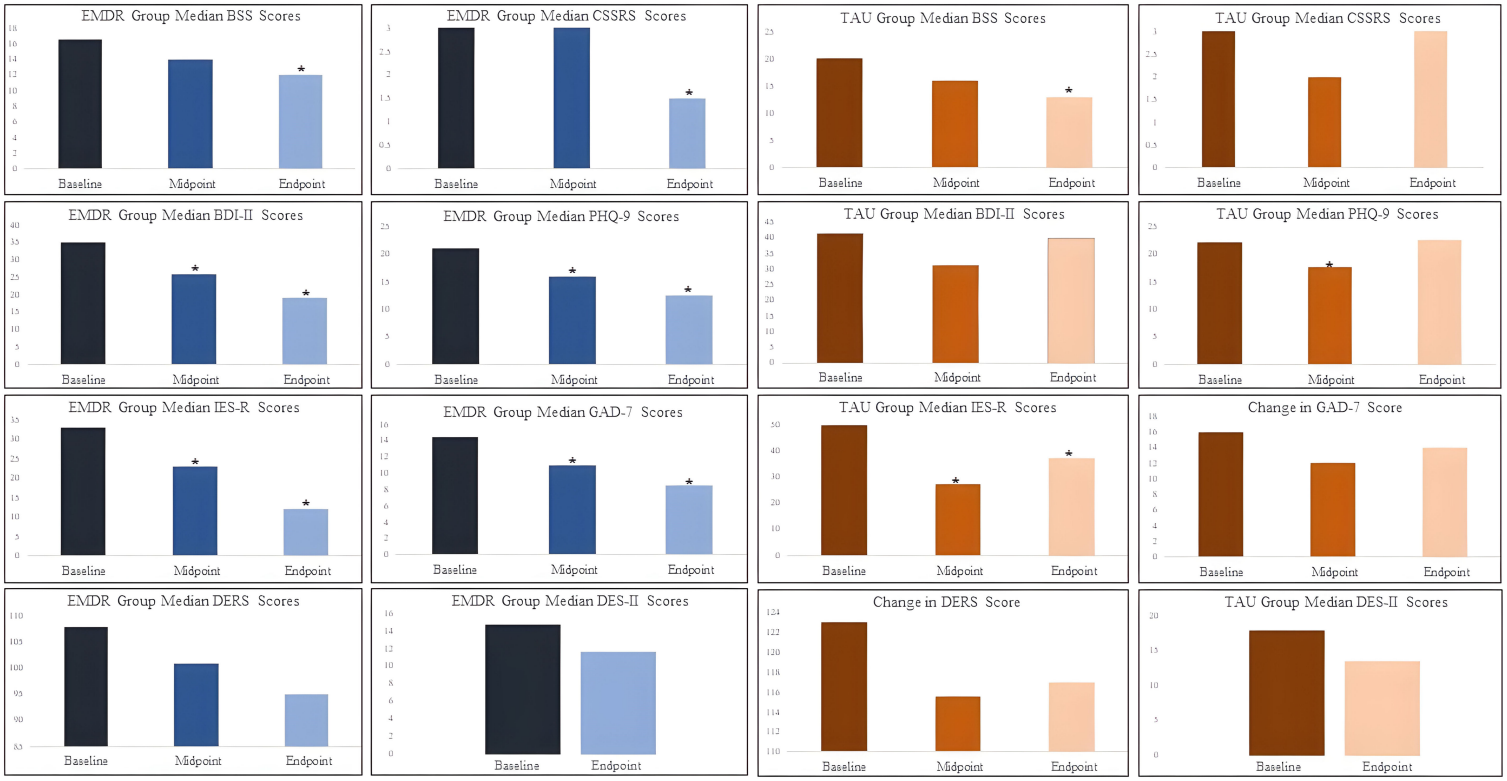
Participant outcome measure scores across time points. *=significant difference from baseline (p≤ 0.05 corrected for multiple comparisons).

Conversely, symptoms in the TAU group appeared to trend downwards from baseline to midpoint, and then worsen thereafter (**Figure 2**). This U-shaped curve, with rebounding symptom scores, may be related to the difference in treatment received by the TAU and EMDR groups in this study. Treatment as usual likely included temporary supportive and stabilization strategies, lessening SI without resolving underlying factors. As mentioned previously, EMDR desensitizes implicit and explicit memories of distressing experiences. In this context, when focused on experiences associated with SI, there may be potential to address repetitive suicidal states, thereby leading to more enduring improvements compared to TAU. Given the distress involved in focusing on such material, the lack of worsening SI in the EMDR group is notable. However, this rationale remains theoretical and speculative at present, awaiting further research to confirm this theory.

We hypothesize that multiple factors may have contributed to an underestimation of the effect of EMDR treatment on SI relative to TAU. Compared to similar studies, the enrolled participants appear to have greater trauma burden and psychiatric complexity. This may have translated into a more extensive memory network contributing to suicidal states. For example, a pilot study by Proudlock and Perris limited inclusion to mental health crises that were clearly linked to a trauma; participants at risk of dissociation were also excluded (14). Fereidouni et al. included a homogeneous population of depressed inpatients with SI but excluded those with previous suicide attempts. In addition, 80% reported single traumas, mostly in adulthood (12). Our study participants, however, reported a high incidence of early onset SI, chronic SI, multiple suicide attempts, BPD or BPD traits and multiple other psychiatric diagnoses. A significant minority reported lifelong existential themes related to their SI, such as “I’ve always felt like I should never have been born”, likely representing a more entrenched belief system. In this context, we believe more than 12 EMDR sessions may be necessary to address enough pathogenic memories to observe a larger difference between EMDR and TAU groups for SI. In addition, there were some participants in the EMDR group who could not utilize EMDR online, as they, for example, worried it would contaminate their “safe space” at home, or they needed the safety of an in-person therapist, diluting results. Nevertheless, the results from our intention to treat analysis indicate promise and warrant further investigation to explore whether different presentations of SI (e.g., trauma-based, depression-based, existential themes) or patient characteristics warrant specific EMDR strategies. Such strategies might also offer a pathway to resolve SI and suicide related beliefs that persist even after successful treatment of MDD, for example (64).

Research interest in using EMDR for complex populations at risk for suicide has grown in the last decade, with promising results. For example, EMDR has been used to treat PTSD in increasingly complex populations, often with high prevalence of SI, demonstrating good safety and efficacy (16, 65). There are also multiple positive studies for treating MDD, a condition frequently present in those with SI (19–21). Additionally, clinicians have developed specific strategies for addressing suicidal ideas, urges, and states in the context of BPD, MDD, or suicidal intrusions (11, 13, 30, 66), which remain to be fully studied, but are increasingly used in clinical practice. This exploration may stem from observed limitations of cognitive strategies, possibly because distress accompanying such conditions can overwhelm cognitive and emotion regulatory capacities, especially in populations with a history of early life stress or significant trauma. This emerging trend could also be attributed to the increasing recognition that EMDR is a transdiagnostic treatment, capable of reducing a range of symptoms associated with multiple diagnoses (12, 67–69). Our study contributes to this body of literature, supporting the potential for transdiagnostic treatments targeting specific symptom clusters (70, 71). This aligns with the recent establishment of the NIMH Research Domain Criteria (RDoC), promoting research that focuses on behavioral and neural systems within mental disorders, transcending traditional diagnostic categories (72).

This study also adds to the emerging literature suggesting that TFPs can be safely delivered in the context of SI, including through remote delivery. Despite the presence of SI at baseline, therapists directly focused on distressing content ordinarily prompting or closely related to the SI while providing treatment in an online milieu. There was no significant overall indication of symptom exacerbation, contrary to earlier speculation, and the EMDR group experienced numerically fewer serious adverse effects and instances of excessive healthcare utilization, such as emergency room visits and hospitalizations, compared to those receiving TAU. Our findings align with studies on PTSD that included participants with SI, showing a general pattern of reduced PTSD, depressive symptoms, and SI (for recent reviews, see 16,24). Our dropout rate was, notably, even lower than in other studies. The twice-weekly schedule may have influenced this, as intensive scheduling (e.g., frequency greater than weekly) has been linked with lower dropout rates (73).

The perception of treatment safety can have real world implications, influencing treatment strategies, such as limiting access to TFPs and overemphasizing stress avoidance. Multiple authors have noted clinicians’ reluctance to use TFPs due to concerns about possible clinical deterioration, including symptom exacerbation and worsening or treatment-emergent SI (23–25). It is noteworthy that symptom exacerbation is reported in 10 to 46% of participants in PTSD studies, although this is apparently unrelated to treatment response and dropout (23, 25, 74–76). Even so, the prevailing practice often involves restricting TFP until SI resolves, with a focus on “stabilization”. This stabilization typically centers on achieving a sense of safety, providing psychoeducation, and teaching skills. This is an area of ongoing debate, with some arguing that this approach leads to unnecessary delays in definitive treatment - for days to years (77–80).

There is evidence suggesting that excessive avoidance of distress may hinder the resolution of trauma-related symptoms, which we posit includes SI. Both emotional engagement and the mounting of a stress response during therapy sessions are linked to positive therapy outcomes (81, 82). Successful TFP has demonstrated improvement in autonomic sympathetic dysregulation (83, 84). According to memory reconsolidation theory, for a long-term memory trace to be changed, the distressing memory must be brought into awareness along with prediction error, resulting in new contextual learning and extinction (35, 85). In simpler terms, changing a traumatic memory requires accessing it while introducing something new or unexpected. This new experience can then be integrated during reconsolidation as new learning. Examples might include experiencing a distressing state or memory while feeling safe with a therapist, or envisioning urges play out imaginally to a place of triumph, resolution, or repair. The Fluid Vulnerability Theory of suicide aligns with this concept, proposing that the suicidal belief system is “potentially amenable to change during periods of activation, [and] activation is critical to treatment progress and success” (51). If true, this has important clinical and academic implications, including for suicide research.

Despite the promising results observed in this study, caution is warranted, particularly for those with severe dissociative symptoms who are at heightened risk for severe suicidal behavior (53). This specific cohort, which comprised one-third of referrals, was intentionally excluded from our study. Engaging in EMDR with that high-risk group presents challenges for many reasons, including extreme difficulty in accessing and tolerating emotion, dissociation hindering emotional learning, severe attachment difficulties impeding therapeutic progress, and scarcity of *adaptive information* from experiences related to safety and self-efficacy due to extensive early and repeated wounding (11, 28, 86, 87). There have also been reported cases of clinical deterioration when applying standard EMDR to severe dissociative disorders, such as Dissociative Identity Disorder (88). This has led to the development of specific, titrated EMDR protocols that incorporate stabilization techniques (89–92). We echo the work of others, underscoring the necessity for further research to precisely identify indicators of readiness and therapy suitability, address risk factors for poor outcomes, optimize preparation, individualize treatment, and offer choices for individuals (78).

### 4.1 Strengths

This study had several strengths. First, the study population sampled a real-world, complex psychiatric population. Second, the study had few exclusion criteria, thereby enhancing its generalizability. Finally, the study protocol allowed for treatment individualization, potentially providing a more realistic representation of how EMDR treatment might be delivered in a real-world clinical setting.

### 4.2 Limitations

Several important limitations must also be acknowledged. First, the sample size was relatively small, and the follow up period lasted only 4 months. Several factors likely contributed to the relatively small sample size, including the onset and consequences of the COVID-19 pandemic, which delayed the project and taxed referral sources. We had initially planned on an in-person design but had to adapt in the face of COVID-19 related precautions. In addition, we believe that the web-based delivery of EMDR treatment impacted recruitment, both at the start of the study, and at the end, when there was an increased desire for in-person care. While the size of our sample precludes wide generalizability of our findings, our preliminary results are encouraging as we observed significant changes despite the clinically complex sample with significant heterogeneity.

Furthermore, only four EMDR participants completed all 12 EMDR sessions prior to the midpoint assessment. The timepoints for repeat measures were fixed to minimize the passage of time as a confounding variable between the two groups. However, factors such as scheduling and COVID-related illness did not allow all participants to finish the active treatment at the same time. A longer follow-up may have allowed further differentiation between groups; previous studies, for example, have shown further improvements over time (93). As discussed earlier, the intervention duration may have been too brief given the prevalence of early and longstanding SI and high trauma burden. This study excluded those with severe dissociative symptoms or a DES-II score greater than 34. Additionally, the study took place during the COVID-19 pandemic which likely imposed unforeseen stress on study participants. It is possible that some participants might have experienced loss or reduction in access to mental health supports, potentially influencing the study’s outcomes.

It is important to note that technical challenges, lack of access to computers, or lack of privacy prevented the enrollment of some individuals, and one individual was unable to continue the treatment due to difficulty tuning into bodily sensations online. Although available research studies point largely to equivalent efficacy for online versus in-person delivery of TFP, this may not hold true for everyone. Further investigation is needed to better delineate the personal factors and environmental barriers that render the online environment unsuitable for some, especially in populations with attachment-related adversity that may be particularly sensitive to such factors.

## 5 Conclusion

This study provides preliminary evidence supporting the safe and effective use of web-based clinician-delivered EMDR as a transdiagnostic treatment aimed at reducing SI and symptoms of depression, anxiety, and posttraumatic stress. To our knowledge, it is also the first study to investigate the effectiveness and safety of web-based EMDR in a real-world, highly complex psychiatric population. While results are encouraging, addressing methodological limitations is needed before considering wider implementation. Further research is also needed to better understand who is or is not suitable for this intervention, including who would benefit from in-person versus web-based treatment. Finally, there is a need for clarity on optimal preparation, treatment duration and intensity.

## Data availability statement

The raw data supporting the conclusions of this article will be made available by the authors, without undue reservation.

## Ethics statement

This study involved human participants and was approved by the Health Research Ethics Board, University of Alberta. This study was conducted in accordance with the local legislation and institutional requirements. The participants provided their written informed consent to participate in this study.

## Author contributions

LB: Conceptualization, Data curation, Formal analysis, Funding acquisition, Investigation, Methodology, Resources, Supervision, Validation, Writing – original draft, Writing – review & editing. SY: Data curation, Formal analysis, Project administration, Writing – original draft, Writing – review & editing. SP: Conceptualization, Methodology, Writing – review & editing. AA: Conceptualization, Methodology, Writing – review & editing. KO: Conceptualization, Methodology, Writing – review & editing, Resources. SB: Writing – review & editing. AG: Conceptualization, Formal analysis, Methodology, Supervision, Writing – review & editing. OW: Conceptualization, Data curation, Formal analysis, Funding acquisition, Investigation, Methodology, Resources, Supervision, Validation, Writing – original draft, Writing – review & editing.
