# Supplementary material for: Randomized controlled trial investigating web-based, therapist delivered eye movement desensitization and reprocessing for adults with suicidal ideation
Source: Front Psychiatry. 2024 Feb 16;15:1361086. doi: 10.3389/fpsyt.2024.1361086 (PMC10904458; doi:10.3389/fpsyt.2024.1361086)
Supplement: Supplementary file 1 [file Table_1.docx]

**Table 1.** Outcome Measure Score Comparisons Between Time Points in EMDR (n=20) and TAU (n=22) Groups.

| **Measure (Group)**  Time Point | **p-value (*significant after correction)** | **Benjamini-Hochberg p-value Correction** |
| --- | --- | --- |
| BSS (EMDR Group) |  |  |
| Baseline compared to Midpoint | 0.395 | 0.05 |
| Baseline compared to Endpoint | 0.009* | 0.017 |
| Midpoint compared to Endpoint | 0.03* | 0.033 |
| CSSRS (EMDR Group) |  |  |
| Baseline compared to Midpoint | 0.226 | 0.05 |
| Baseline compared to Endpoint | 0.004* | 0.017 |
| Midpoint compared to Endpoint | 0.051 | 0.033 |
| IES-R (EMDR Group) |  |  |
| Baseline compared to Midpoint | 0.003* | 0.033 |
| Baseline compared to Endpoint | 0.002* | 0.017 |
| Midpoint compared to Endpoint | 0.08 | 0.05 |
| BDI-II (EMDR Group) |  |  |
| Baseline compared to Midpoint | 0.041* | 0.05 |
| Baseline compared to Endpoint | 0.001* | 0.017 |
| Midpoint compared to Endpoint | 0.005* | 0.033 |
| PHQ-9 (EMDR Group) |  |  |
| Baseline compared to Midpoint | 0.039* | 0.05 |
| Baseline compared to Endpoint | 0.001* | 0.017 |
| Midpoint compared to Endpoint | 0.031* | 0.033 |
| GAD-7 (EMDR Group) |  |  |
| Baseline compared to Midpoint | 0.001* | 0.017 |
| Baseline compared to Endpoint | 0.004* | 0.033 |
| Midpoint compared to Endpoint | 0.295 | 0.05 |
| BSS (TAU Group) |  |  |
| Baseline compared to Midpoint | 0.082 | 0.033 |
| Baseline compared to Endpoint | 0.009* | 0.017 |
| Midpoint compared to Endpoint | 0.33 | 0.05 |
| IES-R (TAU Group) |  |  |
| Baseline compared to Midpoint | 0.005* | 0.017 |
| Baseline compared to Endpoint | 0.007* | 0.033 |
| Midpoint compared to Endpoint | 0.139 | 0.05 |
| PHQ-9 (TAU Group) |  |  |
| Baseline compared to Midpoint | 0.033* | 0.033 |
| Baseline compared to Endpoint | 0.808 | 0.05 |
| Midpoint compared to Endpoint | 0.004* | 0.017 |

Legend: BSS: Beck Scale for Suicide Ideation; BDI-II: Beck Depression Inventory II; CSSRS: Columbia Suicide Severity Rating Scale; PHQ-9: Patient Health Questionnaire 9; GAD-7: Generalized Anxiety Disorder 7; IES-R: Impact of Events Revised. *indicates a significant difference (p≤ 0.05) based on Wilcoxon test and Benjamini-Hochberg p-value Correction.
